# Analysis of the nutritional composition and organization of school meals in the province of Kadiogo in Burkina Faso: challenges and prospects

**DOI:** 10.3389/fnut.2023.1309730

**Published:** 2024-01-29

**Authors:** Ella W. R. Compaoré, Ousmane Ouédraogo, Tiatou Souho, Marcel D. Bengaly, Mamouna P. Simporé, Mamoudou H. Dicko

**Affiliations:** ^1^Laboratoire de Biochimie, Biotechnologie, Technologie Alimentaire et Nutrition (LABIOTAN), Département de Biochimie-Microbiologie, Université Joseph KI-ZERBO, Ouagadougou, Burkina Faso; ^2^Département des SVT, Faculté des Sciences et Techniques, Université de Kara, Kara, Togo

**Keywords:** school meals, canteen, food ration, school-aged children, micronutrients, macronutrients

## Abstract

**Background:**

In the face of food shortages and precariousness, school meals are an effective means of encouraging pupils to attend and stay in school, and of combating nutritional deficiencies. Unfortunately, there are bottlenecks to be identified and resolved.

**Objective:**

Analyzing the composition of meals served to school-age children in primary schools in the province of Kadiogo, while assessing the opinion of school staff on these meals (Burkina Faso).

**Methods:**

A descriptive cross-sectional survey about school meals was carried out during the period from April to May 2019 among school stakeholders in primary schools in five (05) municipalities of the province of Kadiogo.

**Results:**

Insufficient quantity and quality of rations served were recorded in primary schools. The endogenous initiative canteens represented 46.4% of the registered canteens. The promotion of Health-Hygiene-Nutrition (H-H-N) activities in schools encountered difficulties in covering the sanitary needs of school-aged children because unavailability of socio-sanitary infrastructures. School meals consisted of starchy foods and legumes in rural schools and more diversified meals consisting of fruits and vegetables as well as meat and fish in urban schools. In rural municipalities, school meals were insufficient in quantity and quality, while in the urban municipality, macronutrient intakes were in excess with micronutrient intakes largely deficient.

**Conclusion:**

Despite the shortcomings, school officials specified that school meals cover lunch rations, increase school enrolment, and improve school-aged children’ learning capacity.

## 1 Introduction

Like many countries in sub-Saharan Africa, Burkina Faso has begun a demographic transition with an average annual growth rate of 3.1 and 48% of the population is under 15 years of age (1). Although decreasing, the mortality rate is still high, in part, because of the double burden of malnutrition, which mainly affects school-age children. Given the importance of healthy nutrition for young children and its implication for their contribution to societal development, it is important to have nutrition-specific interventions targeting young people (2–4). Such interventions will enable countries in transition, such as Burkina Faso, to benefit in the future from healthy human resources capable of driving sustainable development. Therefore, the challenge is how to make such investments a priority in the basic social sectors including education and health (5). It is in this context that school feeding programs called school canteens are established. These programs consist of serving meals to pupils or giving them food rations to take home. These school canteens operate through institutional school feeding programs implemented by different agencies depending on the region (6). The agencies’ contributions are often complemented by the participation of communities and families, hence the name “endogenous canteens.” The initial objective of school canteens was to make them effective support tools for the improvement of school results and thus to positively impact the quality and performance of the education system as a whole (7). However, in the face of food insufficiency and precariousness, these school meals have very quickly established themselves as effective means of promoting access to and retention in school, but also as a means of developing education and combating nutritional deficiencies (8). School canteens have been in existence for many years and their effect as an enhancer of children’s school attendance and performance has been demonstrated in several countries. However, the importance of canteens in the diet and nutritional status of children remains poorly documented.

Many challenges remain, particularly in terms of organization and mobilization of financial and logistic resources for the optimization of programs. In this regard, one of the bottlenecks is the inability to provide school-aged children with meals sufficient in quantity and quality on a permanent basis throughout the school year given the national scope of the program. In this context, the present study aimed at analyzing the meals served to school-aged children in primary school in the province of Kadiogo in Burkina Faso.

## 2 Methodology

### 2.1 Study site

**Figure 1:**
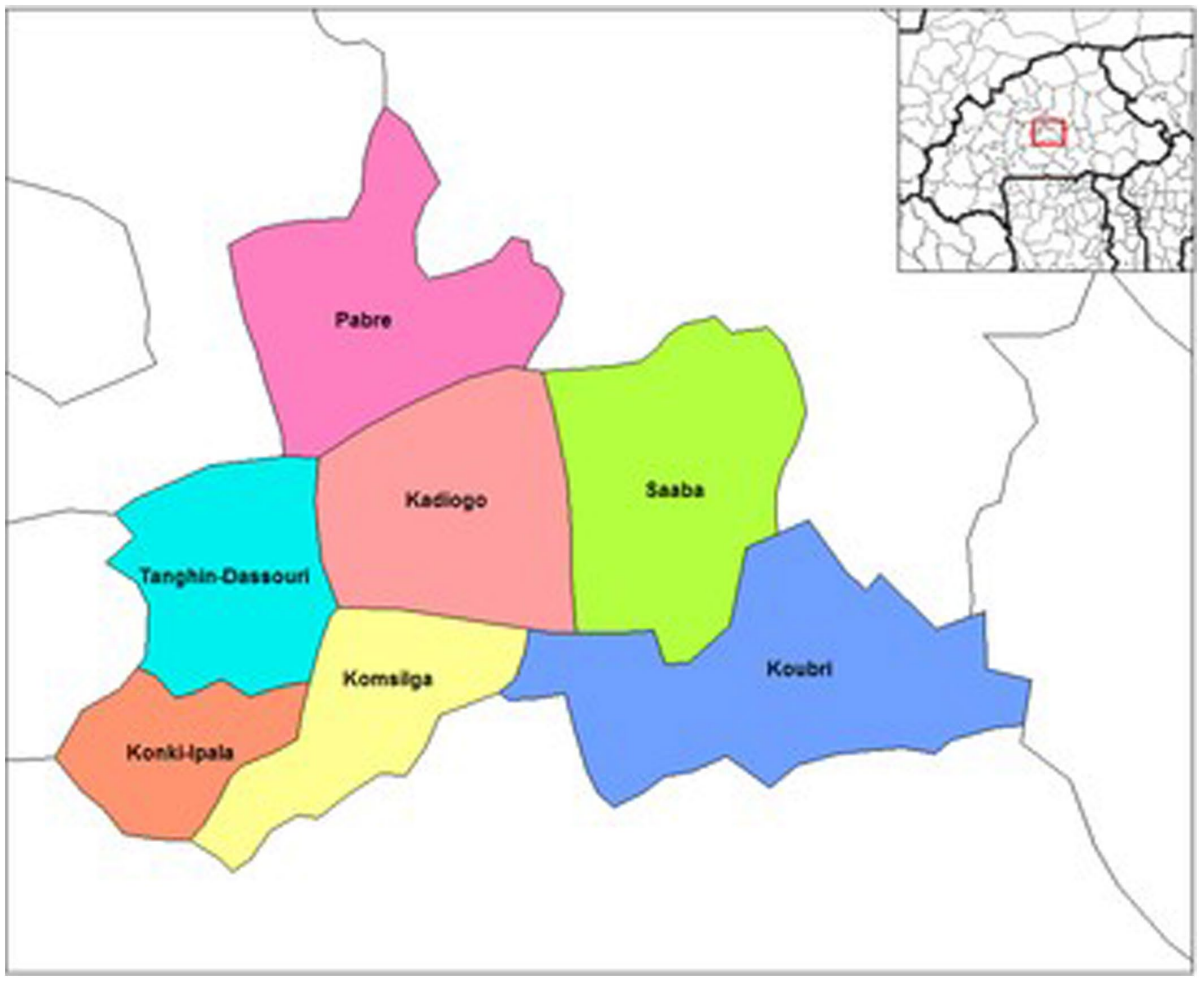
Representative map of the Kadiogo province.

This study was conducted in primary and private schools with school canteens in the province of Kadiogo, specifically in the rural communities of Komki-Ipala, Tanghin Dassouri, Pabré, Saaba, and urban communitie of Ouagadougou (Figure 1).

### 2.2 Study type, period, and population

This is a descriptive cross-sectional study. The study took place over a two-month period, from April to May (2019). The study population was responsible for primary schools. Data collection consisted of recording school officials’ assessments of school meals and analyzing the composition of meals served in public and private primary school canteens selected in Kadiogo province.

### 2.3 Sampling and data collection

The sampling of schools for each municipality was done in Open-Epi. Primary schools were selected on the basis of the availability of a school canteen and in a reasoned manner of school/municipality from all primary schools in the province of Kadiogo. Five municipalities were selected out of the seven municipalities (9) of the province including five schools per municipality (three public schools, two private) schools in the four rural municipalities and 11 schools in the urban municipality (10).

### 2.4 Inclusion criteria

All public and private educational schools with school canteens were considered during the sampling. These canteens must operate on a regular basis and be part of the selection criteria in the province of Kadiogo. Excluded from this study were educational schools that had already exhausted their foodstuff and school officials who refused to participate in the study.

### 2.5 School meals composition analysis method

The food group composition of school meals was analyzed, using the list of food groups suitable for school-aged children as well as food composition tables (11). The nutritional value of the different meals was calculated taking into account the amount of ingredients, the method of preparation and the yield and nutrients retention factors according to Bognar description (12).

### 2.6 Data processing and analysis

The analysis of the data on the school official’ evaluation of school meals was carried out using SPSS version 20.0 software and an appropriate input mask developed for this purpose. The Chi-square test was used to compare the variables. For all tests and comparisons, the significance level was set at *p* ≤ 5%.

## 3 Results

Of 31 schools identified for the study, 28 school officials were interviewed. Schools interviewed included 21 public and 7 private schools. The three schools excluded from the study were schools whose canteens had exhausted their food supplies. Approximately 46.4% of the educational structures surveyed had installed endogenous canteens within their establishment.

### 3.1 Report from school officials

**Figure 2:**
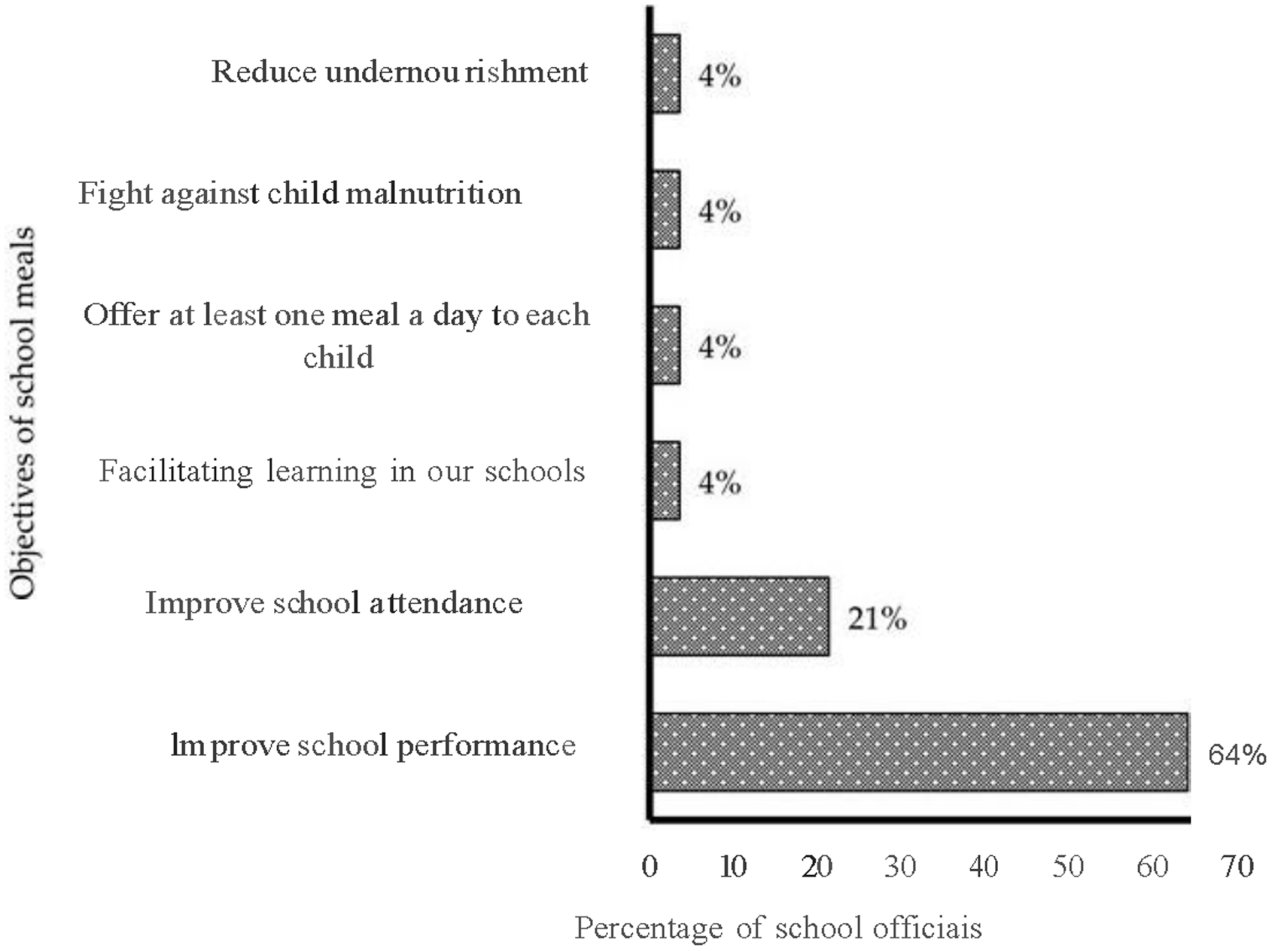
Goals of school meals according to school officials.

The survey of school officials shows that they recognize six objectives for school meals, of which the objectives of improving academic performance and increasing children’s attendance at school ranked first and second (Figure 2).

Regarding food supplies, 64% of officials surveyed reported that the amount of food provided was insufficient to cover school-aged children’ food needs throughout the school year whereas, 29% mentioned that the quantities were average and 7% of them reported that the quantity was sufficient (Table 1).

**Table 1:** Assessment of canteens by school officials.

Estimation of the quantity of food received	*n*	%
Sufficient	2	7.1
Average	8	28.6
Insufficient	18	64.3
Total	28	100.0

### 3.2 Appreciation of canteens regarding the quality of food received

The study found that 64.3% of school canteen cooks rated the physical quality of received food as average and 35.7% of cooks found the food to be of poor quality.

### 3.3 Promotion of health-hygiene-nutrition activities in schools

Only 28.6% of schools had received deworming products for their school-aged children and the majority of schools (71. 4%) had not benefited from it. However, the goal is to combat intestinal worms and some skin diseases in children. With regard to school-based supplementation activities, only 14.3% had been supplemented with vitamin A, and 7.1% with iodine. No school received iron supplementation. It can be established that the majority of school-aged children were not receiving micronutrient supplementation. Twenty-eight point 6 % (28.6%) of educational facilities had a first-aid medical kit and 39.3% were able to sensitize the community on health, hygiene and nutrition activities (Table 2).

**Table 2:** Promotion of Health-Hygiene-Nutrition activities in schools.

Promotion of HHN activities in schools	YES		NO	
	*N*	%	*N*	%
Deworming of school-aged children	8	28.6	20	71.4
Supplementation with vitamine A	4	14.3	24	85.7
Iron supplementation	0	0.0	28	100.0
Iodine supplementation	2	7.1	26	92.9
Medical kit	8	28.6	20	71.4
Sanitation activities	28	100.0	0	0.0
Sensitization on HHN	11	39.3	17	60.7
Participation in competitions on HHN	7	25.0	21	75.0

### 3.4 Composition of school meals

According to the school meals composition by food groups, 9 food groups defined by the FAO (13) for school-age children, only 4 were included in the meals served, namely starchy foods; legumes, nuts and seeds; fruits and vegetables; and meat and fish (Table 3). This was mostly true in the urban municipality. In rural areas, the majority of school-aged children in public schools consumed only 2 groups (cereals and legumes) out of the 9 with the exception of a few private schools that went beyond 03 food groups (adding cereals, legumes, or fish).

**Table 3:** Composition of school meals.

Food group covered by school meals	YES		NO	
	Consumed		Not-consumed	
	*n*	%	*n*	%
Fruits and vegetables	12	42.9	16	57.1
Meat and fish	12	42.9	16	57.1
Legumes, nuts and seeds	28	100.0	0	0.0
Milk and milk products	0	0.0	28	100.0
Starchy food	28	100.0	0	0.0
Other Fruits and vegetables rich in vitamin A	0	0.0	28	100.0
Eggs	0	0.0	28	100.0
Offal	0	0.0	28	100.0
Dark green leafy vegetables	0	0.0	28	100.0

### 3.5 Benefits of school meals for education

85.7% of school officials believe that school meals increased school enrolment and strengthened the relationship with parents. Also 35.7% of the school officials had seen the reality of the endogenous canteen within their schools and 3.6% of the schools had set up a school field which is a garden set up in the schools to improve the provision of balanced meals (Table 4).

**Table 4:** Benefits of school meals in schools.

Benefits of school meals for education	YES		NO	
	*n*	%	*n*	%
Improves school enrollment rate	24	85.7	4	14.3
Improves academic performance	28	100.0	0	0.0
Decreases the number of grade repeats	28	100.0	0	0.0
Decreases the number of dropouts	27	96.4	1	3.6
Improves school completion rate	28	100.0	0	0.0
Improves academic results	28	100.0	0	0.0
Improves nutritional status	28	100.0	0	0.0
Promotion of endogenous canteens	10	35.7	18	64.3
Establishment of school gardens	1	3.6	27	96.5
Strengthens relationships with parents	24	85.7	4	14.3
Ensures the maintenance of children	28	100.0	0	0.0

### 3.6 Comparison of who recommended nutritional requirements to the composition of school meals

During the surveys in the selected schools of the Kadiogo province, only one lunch ration was recorded per day, which was cereal base. Using the nutrient contents per 100 g of school meals, the nutrient contents of the pupils’ lunch ration were calculated according to the recommended scales (150 g of rice, 35 g of beans/mung beans and 16 mL of oil). These nutrient contents of the pupils’ lunch ration were used to estimate daily intakes based on the WHO recommended nutrient requirements. Based on the above results, the energy intake of some rations was above the recommended value. Meals such as Soumbala rice, Jollof rice, and Mung Beans rice contributed 165.8, 161.7, and 182.7% of energy requirements. These types of meals were seen in the urban community with excess energy intakes.

The data also showed that protein intakes are relatively low and lipid intakes were high for all municipalities surveyed. On the other hand, the carbohydrate intakes of Jollof rice (120.2%), soumbala (fermented nera grain) rice (120.9%) and mung beans rice (127.7%) far exceeds the requirement. As a result, the school meals’ content in macronutrient (especially carbohydrates) and energy requirements was above the requirements in the urban municipality. Except for mung beans rice, which contributes 21.7% of phosphorus requirements, and Jollof rice, which contributes 36% of vitamin A requirements, no other type of school meal contributed sufficiently to the micronutrient requirements for those assessed (Table 5).

**Table 5:** Comparison of WHO nutritional requirements to the composition of school meals.

Meal		Couscous beans	Soumbala rice	Jollof rice	Mung beans rice	Rice-beans	Nutritional requirements
Energy	(Kcal)	260.8	707.9	690.6	780.1	260.8	427.0
	%	61.1	165.8	161.7	182.7	61.1	
Protein	(g)	7.9	13	10.8	17.9	7.9	41.0
	%	19.3	31.7	26.3	43.7	19.3	
Lipids	(g)	18.1	19.3	18.4	18.7	18.1	21.3
	%	85	90.6	86.4	87.8	85	
Carbohydrates	(g)	20.2	120.9	120.2	127.7	20.2	100.0
	%	20.2	120.9	120.2	127.7	20.2	
Calcium	(mg)	30	59.7	61.5	40.3	30.0	800.0
	%	3.8	7.5	7.7	5.0	3.8	
Magnesium	(mg)	0.0	0.0	0.0	49.4	0.0	130.0
	%	0.0	0.0	0.0	38	0.0	
Iron	(mg)	2.3	3.8	3.0	4.6	2.3	17.8
	%	12.9	21.3	16.9	25.8	12.9	
Phosphorus	(mg)	0.0	0.0	0.0	108.6	0.0	500.0
	%	0.0	0.0	0.0	21.7	0.0	
Vitamin A	(μg E.R)	4.2	0.0	143.9	0	4.2	400.0
	%	1.1	0.0	36.0	0	1.1	
Vitamin C	(mg)	0.2	0.0	1.5	0.6	0.2	25.0
	%	0.8	0.0	6.0	2.4	0.8	

## 4 Discussion

School officials in this study noted that through the implementation of school canteens, school-aged children’ academic performance has improved significantly. The enrolment rate of children has increased considerably since the introduction of the school feeding program in public and private schools. School meals would also be a means of combating malnutrition among school-aged children. The objectives of school meals cited by principals also contribute to the achievement of the objectives set by WHO (14) for school meals, which included improving school enrolment, attendance and performance WHO (14).

The quantity of food received in the schools surveyed was insufficient because the allocation lasted only three months out of nine (09) during the entire school year. These shortcomings are often made up for by the presence of an endogenous canteen in the schools. The adequacy of school meals in schools was conditioned by the interventions of certain non-governmental organisations (NGOs). In any case, the operation of the canteens is part of the administrative framework of Burkina Faso education system, which is decentralized at all territorial levels (15). The government’s decision to transfer the management of school canteens to the communities contributes to the strengthening of the implementation of full communalization and the responsibility of communities for economic development at the grassroots level.

To solve this problem of inadequacy, opinion leaders in the municipalities have developed initiatives at the local level to mobilize resources for the permanent operation of school canteens within the various schools. Also, the irregularity of the allocations was one of the reasons for the organization of the endogenous canteens. To remedy this, it will be necessary to provide schools with food at the beginning of the year in order to improve the implementation of endogenous canteens. OXFAM’s 2015 report stated that irregular allocations and delays in delivery were holding back the program. The complexity of the logistics required to supply all schools in the country on time, particularly in the state-run provinces, the magnitude of the program and the limited resources allocated to it leads to dysfunctions resulting in delays in deliveries. This makes it difficult to organize and plan school canteens and establish endogenous canteens (16).

The quantity was insufficient, but the quality was also lacking, and this was explained by the fact that the food was already damaged on delivery, especially the beans whose quality left something to be desired despite the checks carried out before delivery. A study on social protection and food security in Burkina Faso in an Oxfam report in 2015 indicated that 54% of the surveyed canteens had judged the products distributed in school canteens to be of poor quality (16).

The promotion of Health, hygiene, and nutrition (H-H-N) actions and their implementation encountered difficulties in their implementation because of the lack of support and the actions that were implemented were not sufficient to cover the health needs of school-aged children because of the lack of availability of some socio-sanitary infrastructures in the communities.

In the selected public education facilities in Kadiogo province, only one ration (rice-beans) was documented per day at lunch, which consisted of a cereal base. And this ration contributes to only 61.1% of the school-aged children daily nutritional energy requirements based on daily intakes to ensure normal nutrition in a healthy school-aged children (17). According to FAO, a daily consumption of two food groups could not cover the dietary needs of school-aged children especially in micronutrients while for a number of school-aged children especially in rural areas, school meals should be a privileged source of nutrients essential for their growth and psychomotor development. In most cases, the dietary diversification of school meals remains a challenge to be met in all municipalities of Kadiogo. In rural communities, school meals are insufficient in quantity and quality; micronutrient intakes were largely deficient. In view of the above results, vitamin A supplementation is necessary unless household consumption can cover this deficit because micronutrient deficiencies have profound effects on the health of the brain, bones and the body in general.

The presence of school meals contributed to improved academic performance, retention of school-aged children in school, completion rates and reduced dropouts. In 2013, in Ghana, a study on the school feeding program corroborated the benefits cited above by the principals of the schools surveyed. The implementation of this program has increased enrolment in primary schools in Ghana (18).

## 5 Conclusion

This study highlighted the realities of school nutrition in Burkina Faso. The study showed that school meals were about the same, neither balanced nor varied in most primary schools and that the coverage of school-aged children’ micronutrient nutritional needs was deficient. Despite the realities of school feeding, the results of the study show that school meals play an important role in education as they reduce hunger, drop-outs, repetition rates, increase enrolment, attendance, academic performance, improve attention, school-aged children’ learning capacity and school completion rates. School meals made it possible to provide at least one meal a day. In view of the results, we suggest that further descriptive cross-sectional studies be conducted to better assess school-aged children’ diets at the household and school levels in order to assess their daily consumption in general.

## Data availability statement

The datasets presented in this study can be found in online repositories. The names of the repository/repositories and accession number (s) can be found below: the database used in this study can be provided by the corresponding author upon reasoned request.

## Ethic statement

The study required the consent of the inspectors of the municipalities, the Directors of primary schools, rural and urban communities as well as the managers of school canteens and the cooks.

## Author contributions

EC: Conceptualization, Investigation, Methodology, Supervision, Writing – original draft, Writing – review & editing. OO: Conceptualization, Formal analysis, Investigation, Methodology, Software, Supervision, Writing – original draft, Writing – review & editing. TS: Writing – original draft, Writing – review & editing. MB: Writing – original draft, Writing – review & editing. MS: Conceptualization, Data curation, Formal analysis, Funding acquisition, Investigation, Methodology, Project administration, Resources, Software, Validation, Writing – original draft, Writing – review & editing. MD: Funding acquisition, Writing – original draft, Writing – review & editing.
